# Multi-omics profiling identifies M1 macrophage polarization-associated biomarkers in hepatitis B virus-related acute-on-chronic liver failure

**DOI:** 10.3389/fmicb.2025.1630042

**Published:** 2025-09-24

**Authors:** Chengxi Sun, Cheng Li, Jianguo Hong, Wencheng Lv, Zeyang Liu, Haochen Wang, Qihao Dong, Helgi B. Schiöth, Shuai Gao

**Affiliations:** ^1^Department of Clinical Laboratory, Qilu Hospital of Shandong University, Jinan, China; ^2^Department of Radiology, Qilu Hospital of Shandong University, Jinan, China; ^3^Department of General Surgery, Qilu Hospital of Shandong University, Jinan, China; ^4^Department of Radiology, Jiaozhou Branch of Shanghai East Hospital, Tongji University, Qingdao, China; ^5^Department of Organ Transplantation, Qilu Hospital of Shandong University, Jinan, China; ^6^Department of General Surgery, The First Affiliated Hospital of Shandong First Medical University, Jinan, China; ^7^Department of Surgical Sciences, Functional Pharmacology and Neuroscience, Uppsala University, Uppsala, Sweden; ^8^Department of Hepatology, Qilu Hospital of Shandong University, Jinan, China; ^9^Hepatology Institute of Shandong University, Jinan, China

**Keywords:** multi-omics profiling, hepatitis B virus-related acute-on-chronic liver failure, macrophage polarization, biomarkers, regulatory network

## Abstract

**Background and aims:**

M1 macrophage polarization is essential for the progression of hepatitis B virus-related acute-on-chronic liver failure (HBV-ACLF). In this study, we aim to identify and validate M1 polarization-associated biomarkers to elucidate pathogenic mechanisms and identify potential therapeutic targets for HBV-ACLF.

**Methods:**

Multi-omics data from public databases were analyzed using a co-expression network and two differential expression analyses. Biomarkers were identified by machine learning, ROC curves, and experimental validation. A nomogram was developed to assess the diagnostic efficacy of the biomarkers. Subsequent analyses included functional enrichment, regulatory network construction, computational drug prediction, and molecular docking analysis. Finally, biomarker expression was validated using reverse transcription-quantitative PCR (RT-qPCR) in clinical specimens.

**Results:**

CDC20, CXCL14, FCGR2B, HKDC1, and GPBAR1 were identified as diagnostic biomarkers for HBV-ACLF. The constructed nomogram showed strong diagnostic performance. Functional enrichment analysis revealed multiple pathways enriched in these biomarkers, including tryptophan metabolism and cofactor biosynthesis, etc. Subsequently, a lncRNA-miRNA-mRNA regulatory network was constructed, with key interactions such as XIST/hsa-miR-296-3p/CXCL14 and SNHG14/hsa-miR-510-5p/CXCL14. Further analysis identified multiple drugs associated with the biomarkers, including cholic acid, deoxycholic acid (GPBAR1-targeting agents). Molecular docking revealed favorable binding affinities between the predicted drugs and their targets, for example, cholic acid exhibited a binding free energy of −7.5 kcal/mol with GPBAR1. In validation experiments, RT-qPCR confirmed significant upregulation of all five biomarkers in HBV-ACLF patients compared with healthy controls (HCs).

**Conclusion:**

This study identifies CDC20, CXCL14, FCGR2B, HKDC1, and GPBAR1 as M1 polarization-associated biomarkers, revealing their roles in immune-metabolic dysregulation and proposing novel therapeutic strategies for HBV-ACLF.

## 1 Introduction

Hepatitis B virus-related acute-on-chronic liver failure (HBV-ACLF) is a life-threatening clinical syndrome characterized by rapid hepatic decompensation in patients with pre-existing chronic hepatitis B (CHB), leading to multi-organ failure and high short-term mortality (Zhao et al., 2025). Globally, HBV-ACLF accounts for a significant proportion of liver-related morbidity and mortality (Zhang et al., 2023). The pathogenesis of HBV-ACLF involves a complex interplay of viral reactivation, immune hyperactivation, and dysregulated inflammatory cascades, which drive hepatocyte necrosis and impair hepatic regeneration.

Current diagnostic criteria mainly rely on clinical scores (e.g., MELD, CLIF-C ACLF, and COSSH-ACLF) and nonspecific biomarkers, while therapeutic strategies are limited to liver transplantation and supportive care (Luo J. et al., 2023). The MELD score is more suitable for long-term prognosis assessment compared to the CLIF-C ACLF and COSSH-ACLF score. Meanwhile, based on the complicated assessment of organ failure, the CLIF-C ACLF and COSSH-ACLF score still need to be simplified and more accurate (Choudhury et al., 2025). Traditional nonspecific markers such as INR and TBIL can also reflect the severity of HBV-ACLF, but their role in predicting disease prognosis and identifying new therapeutic targets remains limited. Therefore, there is an urgent need to identify novel biomarkers that reflect disease progression and offer therapeutic potential (Li et al., 2021).

Macrophages, as central mediators of innate immunity, exhibit remarkable plasticity and participate in the development of various liver diseases (Luo S. et al., 2023). In HBV-ACLF, excessive M1 polarization contributes to amplified hepatic inflammation through the secretion of pro-inflammatory cytokines (e.g., TNF-*α*, IL-6) and reactive oxygen species (Taru et al., 2024). The G protein-coupled bile acid receptor 1 (GPBAR1, also known as TGR5), a bile acid-sensitive receptor, has emerged as a key regulator of metabolic and inflammatory processes (Biagioli et al., 2023). Recent studies showed that GPBAR1 could modulate macrophage polarization (Liu et al., 2025). GPBAR1 activation has been linked to both hepatoprotective and pro-fibrotic effects, highlighting its context-dependent functions (Shi et al., 2020). Biagioli et al. reported that the small molecule CHIN117, which functions as a GPBAR1 agonist and CYSLTR1 antagonist, effectively reversed acetaminophen-induced drug-induced liver injury (DILI) in mice, demonstrating that GPBAR1 play a significant hepatoprotective role in liver injury (Biagioli et al., 2023). However, the mechanistic interplay between GPBAR1 signaling, macrophage polarization, and HBV-ACLF progression remains unexplored. Investigation of this regulatory axis might lead to the identification of novel therapeutic approaches.

In this study, we employed an integrative multi-omics bioinformatics approach with experimental validation to identify and validate biomarkers associated with macrophage polarization and GPBAR1 signaling in HBV-ACLF. This approach may elucidate the underlying mechanisms of immune-metabolic dysregulation and offer novel therapeutic strategies for HBV-ACLF.

## 2 Materials and methods

### 2.1 Data preparation and samples collection

The GEO database^1^ provided the training and validation sets for HBV-ACLF. The training set GSE38941 (platform: GPL570) included transcriptome data from 17 HBV-ACLF and 10 control human liver tissue samples (Nissim et al., 2012). The validation set GSE14668 (platform: GPL570) contained transcriptome data from 8 HBV-ACLF and 8 control human liver tissue samples (Farci et al., 2010). To confirm biomarker expression, we included 9 human liver samples comprising 5 healthy controls (HCs) and 4 HBV-ACLF patients. Liver samples from HCs were obtained from patients without underlying chronic liver diseases who underwent partial hepatectomy for hepatic hemangioma. Adjacent normal liver tissues were obtained from the resected lesions. Liver samples from HBV-ACLF patients were obtained during liver transplantation. HBV-ACLF was diagnosed according to the APASL consensus recommendations: (1) presence of serum hepatitis B surface antigen (HBsAg) for >6 months; (2) progressive jaundice (serum bilirubin ≥5 mg/dL); (3) coagulopathy (INR ≥ 1.5 or prothrombin activity <40%) (Sarin et al., 2019). Exclusion criteria included: (1) co-infection with human immunodeficiency virus (HIV), hepatitis A, C, D, or E virus, Epstein–Barr virus, or cytomegalovirus; (2) other liver diseases such as alcoholic hepatitis or autoimmune liver diseases; (3) liver cancer.

The study protocol was approved by the Institutional Research and Ethics Committee of Qilu Hospital, Shandong University, and other participating centers, in accordance with the 1975 Declaration of Helsinki guidelines. Written informed consents were obtained from all patients.

### 2.2 Acquisition of key module genes

In GSE38941, the infiltration abundances of 10 immune cell types in HBV-ACLF and control samples were evaluated using the quanTIseq algorithm. The Wilcoxon test was applied to identify differential immune cells by comparing infiltration abundances between the two groups (*p* < 0.05). Using M1 macrophages as the trait, WGCNA was performed with the WGCNA package (v1.73) (Langfelder and Horvath, 2008) to identify genes significantly associated with M1 macrophages.

First, the goodSamplesGenes function was used to cluster all samples in GSE38941, and outlier samples were removed. The soft threshold (*β*) was determined using the pickSoftThreshold function. When the mean connectivity approached 0 and *R*^2^ > 0.80, the optimal soft threshold was selected. A co-expression matrix was constructed with a minimum of 200 genes per module. A hierarchical clustering dendrogram was generated using the dynamic tree cut algorithm, with modules represented by distinct colors. Spearman correlation analysis between gene modules and M1 macrophages was performed using the psych package (v2.4.6.26) (|r| > 0.3, *p* < 0.05; Robles-Jimenez et al., 2021). The two modules showing the strongest positive and negative correlations with M1 macrophages were designated as key modules, and their genes were defined as key module genes.

### 2.3 Differential expression analysis

Differential expression analysis was performed in GSE38941 to identify differentially expressed genes 1 (DEGs1) between HBV-ACLF and control samples (adj. *p* < 0.05, |log₂FC| > 1). The Wilcoxon test was used to compare GPBAR1 expression between groups (*p* < 0.05). HBV-ACLF samples were stratified by median GPBAR1 expression into high- and low-expression groups. DEGs2 were identified by comparing these groups (|log₂FC| > 1, adj. *p* < 0.05). Volcano plots and heatmaps were generated using ggplot2 (v3.5.1) (Gustavsson et al., 2022) and pheatmap package (v1.0.12) (Gu and Hübschmann, 2022), respectively.

### 2.4 Identification and analyses of candidate genes

The VennDiagram package (v 1.7.3) (Chen and Boutros, 2011) was used to intersect DEGs1, DEGs2, and key module genes to identify candidate genes. GO and KEGG enrichment analyses (*p* < 0.05) were performed using clusterProfiler package (v 4.15.0.3) (Wu et al., 2021). A protein–protein interaction (PPI) network (confidence score >0.4) was constructed via STRING^2^ and visualized using the Cytoscape package (v3.9.1) (Shannon et al., 2003).

### 2.5 Identification of biomarkers

LASSO regression (glmnet v4.1.4; Engebretsen and Bohlin, 2019) with 10-fold cross-validation was applied to candidate genes. Feature genes were selected at the minimal lambda value where coefficients were non-zero. The receiver operating characteristic (ROC) curve analysis (pROC v1.18.5; Robin et al., 2011) in GSE38941 and GSE14668 identified key genes (AUC > 0.85 in both datasets). Biomarkers were defined as genes with consistent expression trends and significant differences (Wilcoxon test, *p* < 0.05) between HBV-ACLF and controls in both datasets.

### 2.6 Construction and evaluation of nomogram

In GSE38941, a nomogram was constructed using the rms package (v 6.8.1; Xu et al., 2023) to examine the prediction ability of biomarkers for HBV-ACLF. Calibration curve was then created to assess the prediction accuracy.

### 2.7 Enrichment analysis, construction of gene–gene interaction (GGI) network, and chromosomal localization

Spearman correlation study between the biomarkers and other genes in the GSE38941 was conducted through the psych package (v 2.4.6.26; Robles-Jimenez et al., 2021). The correlation coefficient was employed to order the genes from greatest to smallest. The gseKEGG function of the clusterProfiler package (v 4.15.0.3) (Wu et al., 2021) was then applied to perform GSEA analysis, with significant criterion of FDR < 0.25, |NES| > 1, and *p* < 0.05. The biomarkers were then uploaded to the GeneMANIA database^3^ to generate a GGI network, which allowed researchers to investigate genes that shared comparable activities with the biomarkers and the functions they were involved in. Furthermore, the localization of biomarkers on different chromosomes was analyzed by the RCircos package (v 1.2.2) (Zhang et al., 2013).

### 2.8 Regulatory network analysis

Transcription factors (TFs) regulating the biomarkers were predicted in the Encyclopedia of DNA Elements (ENCODE) database^4^ within the NetworkAnalyst platform.^5^ The TF-mRNA regulatory network was constructed. The miRWalk database^6^ was then employed to predict miRNAs. The miRNAs targeting two biomarkers simultaneously were selected to construct miRNA-mRNA regulatory networks. The starBase database^7^ was then employed to forecast lncRNAs based on miRNAs. Additionally, a lncRNA-miRNA-mRNA regulatory network was created. The regulatory networks mentioned above were shown using the Cytoscape package (version 3.9.1) (Shannon et al., 2003).

### 2.9 Disease prediction, drug prediction, and molecular docking

The Comparative Toxicogenomics Database (CTD)^8^ was applied to forecast diseases linked to biomarkers, and a disease-mRNA network was established. Potential drugs that target the biomarkers were then predicted through the DrugBank database.^9^ A drug-mRNA network was also formed at the same time. The regulatory networks mentioned above were shown using the Cytoscape softwore (version 3.9.1) (Shannon et al., 2003). The ability of biomarkers to bind to possible drugs was then investigated by molecular docking analysis. In particular, molecular docking research was done to investigate the binding ability between biomarkers and possible medications. The PDB database^10^ provided the 3D structures of the proteins that corresponded to the biomarkers, while PubChem^11^ provided the 3D structures of the medications. Molecular docking analysis was carried out on the AutoDock Vina website,^12^ and the results were visualized using PyMOL software (v 2.5) (Seeliger and de Groot, 2010).

### 2.10 Reverse transcription quantitative polymerase chain reaction

Total RNA was extracted from tissue samples using TRIzol® Reagent (Invitrogen, United States) following the manufacturer’s protocol. First-strand cDNA was synthesized using PrimeScript™ RT Reagent Kit (Takara). Primers for CDC20, CXCL14, FCGR2B, HKDC1, and GPBAR1 were designed via Primer-BLAST and synthesized by Sangon Biotech. GAPDH served as the internal control. The qRT-PCR reactions were performed in triplicate using SYBR® Green PCR Master Mix (Bio-Rad, United States) on a CFX96 Real-Time PCR System (Bio-Rad). Cycling conditions: 95 °C for 3 min (initial denaturation), followed by 40 cycles of 95 °C for 10 s (denaturation) and 60 °C for 30 s (annealing/extension). Relative gene expression was calculated via the 2^−ΔCt^ method. Statistical significance was assessed by Student’s t-test (*p <* 0.05). Graphpad Prism (v 5.0) was used for graphing and statistics (Baziyar et al., 2024). Detailed primers and sequences were shown in Table 1.

**Table 1:** Primers and sequences of CDC20, CXCL14, FCGR2B, HKDC1 and GPBAR1.

Gene	Primer sequence (5′-3′)
CDC20-F	AATGTGTGGCCTAGTGCTCC
CDC20-R	AGCACACATTCCAGATGCGA
CXCL14-F	ATCACCACCAAGAGCGTGTC
CXCL14-R	CTTCTCGTTCCAGGCGTTGT
FCGR2B-F	TCCAAGAAATTTTCCCGTTCG
FCGR2B-R	CTATGTTTCCTGTGCAGTGGT
HKDC1-F	CCTCAGTACCCAAAACGCCT
HKDC1-R	GACAGGAGGAAGCGGACATC
GPBAR1-F	CGCTACATGGCAGTCCTGAG
GPBAR1-R	GGTAGGGGGCTGGGAAGATA

### 2.11 Statistical analysis

R (v 4.2.2) was utilized to conduct statistical analysis. Difference analysis between groups was executed via the Wilcoxon test (*p* < 0.05). In the RT-qPCR investigations, the t test was implemented to compare the differences between the two groups. A two-tailed *p* value less than 0.05 was considered statistically.

## 3 Results

### 3.1 The 2,796 key module genes were determined

**Figure 1:**
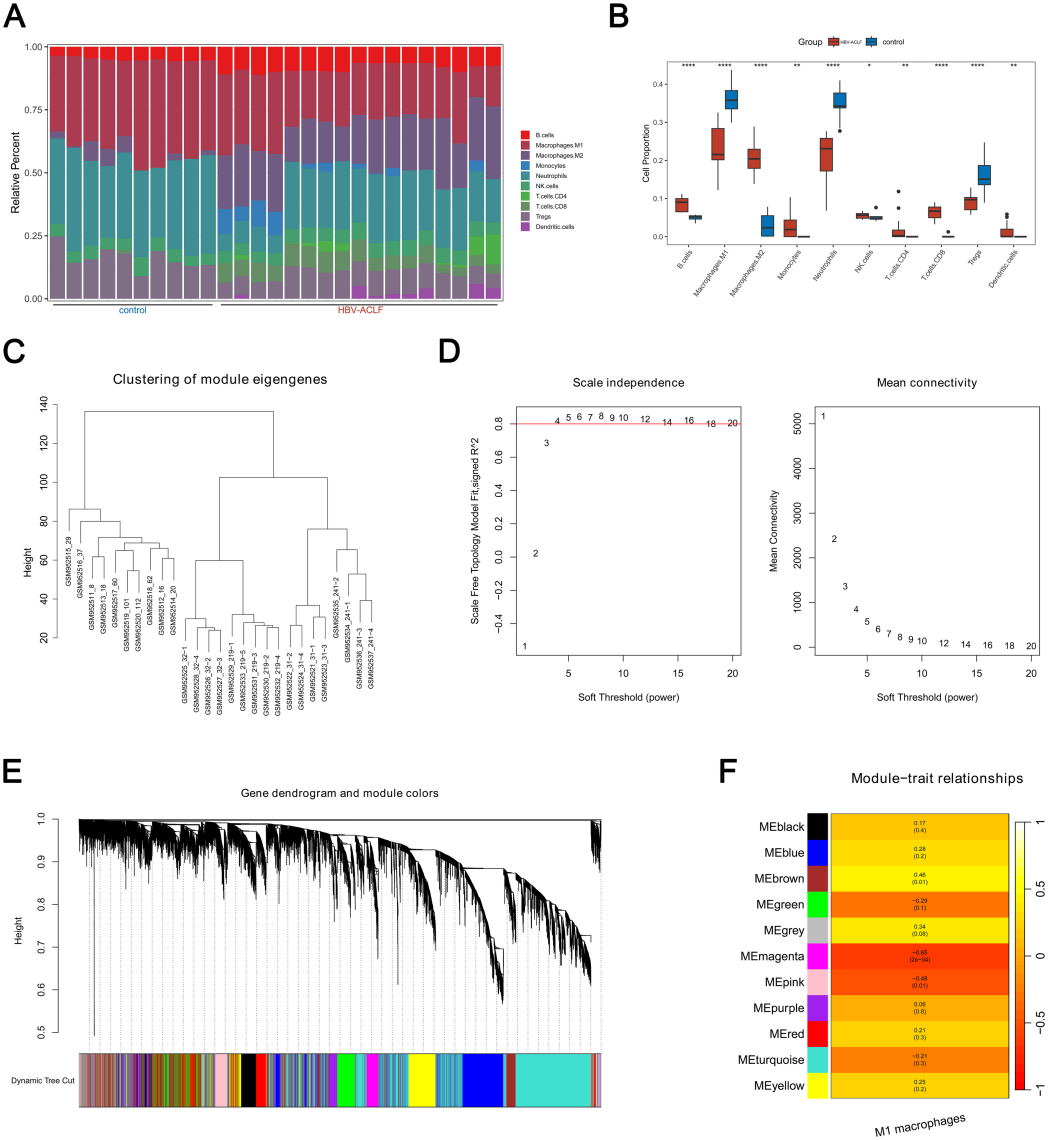
2,796 key module genes in HBV-ACLF and control samples were determined in the GSE38941 dataset. **(A)** The infiltration abundance of ten immune cells in HBV-ACLF and control samples (*p* < 0.05). **(B)** Analysis of the abundance of immune cell infiltration between HBV-ACLF and control samples by Wilcoxon rank sum test (*p* < 0.05). **(C)** WGCNA analysis to detect outlier samples in the GSE38941 dataset. **(D)** Screen the scale-free network to select the optimal soft-threshold power (*R*^2^ > 0.8, mean connectivity < 200). **(E)** 11 gene modules were chosen from the Gene Hierarchical Clustering Dendrogram. **(F)** Correlations between distinct gene modules and M1 macrophages.

Figure 1A displayed the infiltration abundance of 10 immune cells in both the HBV-ACLF and control samples in the GSE38941 dataset. The infiltration abundance of the remaining nine immune cells, with the exception of NK cells, was then interestingly found to differ significantly between HBV-ACLF and control samples (*p <* 0.05). For example, M1 macrophages had larger infiltration abundance in control samples (Figure 1B). Afterwards, WGCNA was performed. In GSE38941, no outlier samples were detected (Figure 1C). When the mean connectivity was near 0 and the R^2^ value was larger than 0.80, the ideal soft threshold *β* was found to be 9 (Figure 1D). The 11 gene modules were then chosen (Figure 1E). The MEmagenta module showed the strongest negative correlation (cor = −0.65, *p =* 2 × 10^−4^) with M1 macrophages, while the MEbrown module showed the strongest positive correlation (cor = 0.46, *p =* 0.01; Figure 1F). As a result, 2,287 genes from the MEbrown module and 509 genes from the MEmagenta module were determined to be key module genes, amounting to a total of 2,796 genes.

### 3.2 The 34 candidate genes were acquired

**Figure 2:**
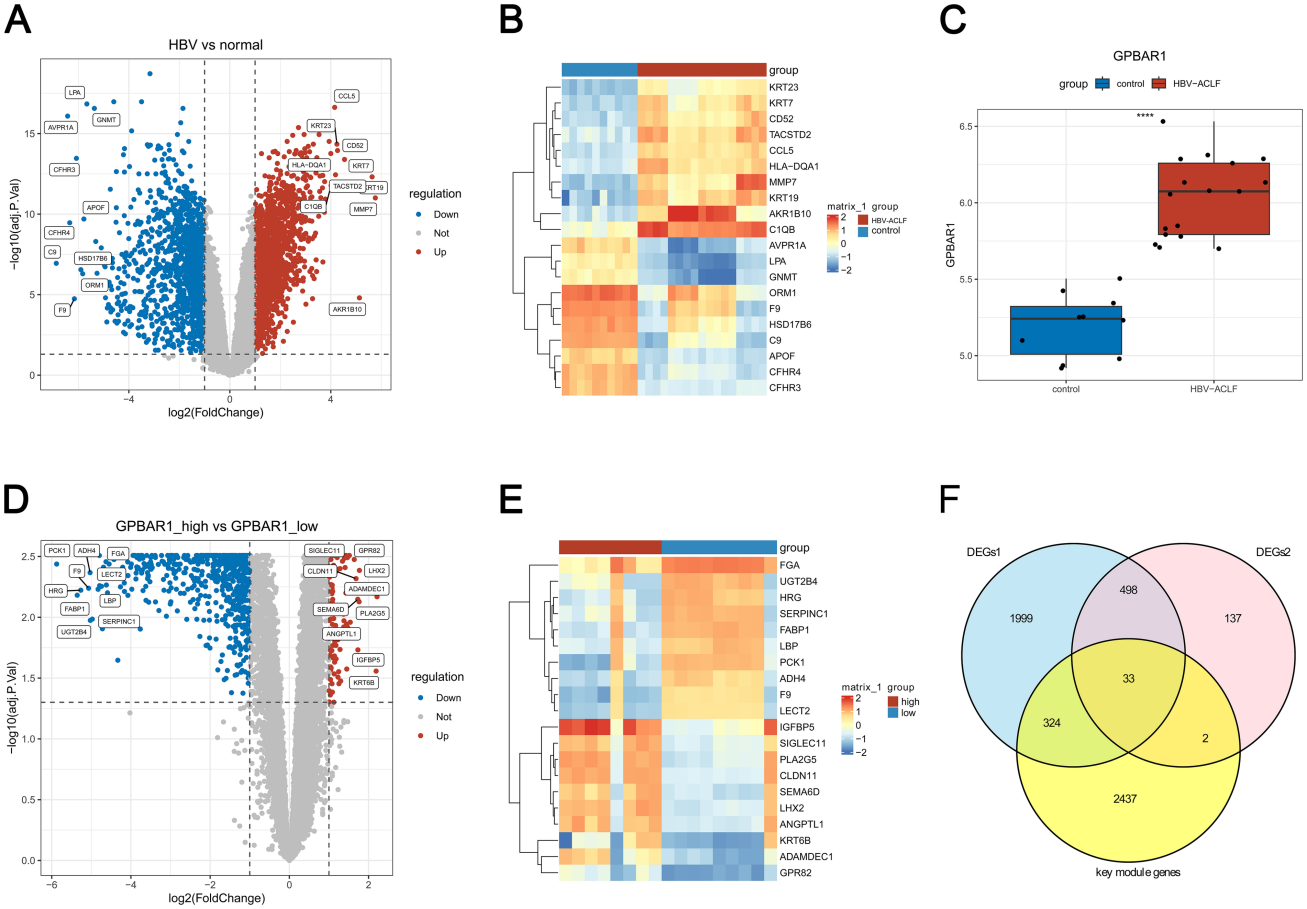
Identification of candidate genes associated with M1 macrophages and GPBAR1 in HBV-ACLF. **(A)** Differentially expressed genes 1 (DEGs1) between HBV-ACLF and control samples (Adj. *p* < 0.05, |log_2_FC| > 1) **(B)** The expression of the top 10 genes that were up- and down-regulated in HBV-ACLF and control samples. **(C)** The expression levels of GPBAR1 between the HBV-ACLF and control samples (*p* < 0.05). **(D)** The up-regulated genes and down-regulated genes in GPBAR1 high and low expression patients. **(E)** The expression levels of top 10 genes between GPBAR1 high and low expression patients. **(F)** Venn diagram highlighting differentially expressed genes.

By differential expression analysis, 2,854 DEGs1 were identified between HBV-ACLF and control samples in GSE38941. In HBV-ACLF samples, 1,601 genes were up-regulated and 1,253 genes were down-regulated. In the volcano plot, the top 10 up- and down-regulated genes were labeled from highest to lowest in order of |log_2_FC| (Figure 2A). The heatmap (Figure 2B) showed the expression of the top 10 genes that were up- and down-regulated in the HBV-ACLF and control samples (Figure 2B). Figure 2C showed a significant difference (*p <* 0.05) in GPBAR1 expression levels between the HBV-ACLF and control samples. The median value (6.07) of GPBAR1 expression was then used to separate the groups with high and low expression. 670 DEGs2 were found between the high and low expression groups. In the high expression group, there were 90 up-regulated genes and 580 down-regulated genes (Figures 2D,E). After that, 2,796 key module genes, 2,854 DEGs1, and 670 DEGs2 were taken for intersection and 33 genes were acquired (Figure 2F). Because these 33 genes did not contain GPBAR1, both the above 33 genes and GPBAR1 were included as candidate genes for subsequent analysis.

### 3.3 Candidate were involved in multiple pathways

The 34 candidate genes were significantly associated with 442 GO terms and 7 KEGG pathways (*p <* 0.05). The 442 GO terms included 366 BPs, 30 CCs, as well as 46 MFs. Ranked by *p*-value from smallest to largest, the top 10 terms for BPs, CCs, and MFs were shown separately, including nuclear division, meiotic spindle, histone kinase activity, and so on (Supplementary Figure 1A; Supplementary Table 1). Similarly, 7 KEGG pathways included oocyte meiosis, cell cycle, and so on (Supplementary Figure 1B; Supplementary Table 2). Moreover, a PPI network was created. Among them, genes such as DEPDC1 and ASPM had a relatively strong interaction with other genes (Supplementary Figure 1C).

### 3.4 The 5 biomarkers were determined

**Figure 3:**
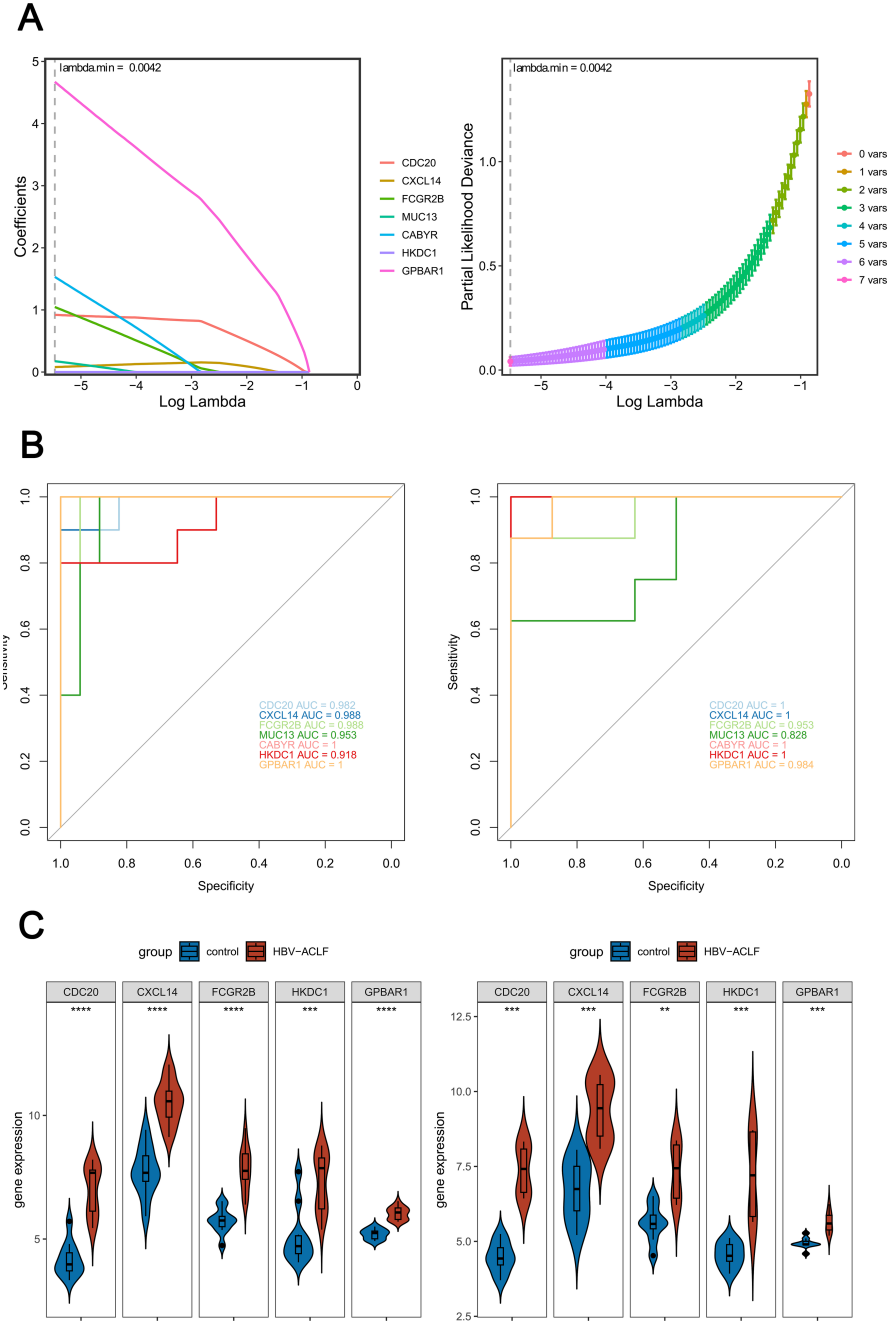
Screening key genes by machine learning. **(A)** 7 feature genes (CDC20, CXCL14, FCGR2B, MUC13, CABYR, HKDC1, GPBAR1) were identified by LASSO regression analysis (lambda.min = 0.0042). **(B)** ROC curve analysis on key genes in HBV-ACLF samples and control samples was performed by calculating the area under the curve (AUC) values (AUC > 0.85, AUC ≠ 1). Left: Training set GSE38941; Right: Validation set GSE14668. **(C)** Expression levels of potential biomarkers in the training set and validation set (Wilcoxon Rank-Sum Test, *p* < 0.05).

In the LASSO regression analysis, 7 feature genes (CDC20, CXCL14, FCGR2B, MUC13, CABYR, HKDC1, GPBAR1) were identified when lambda.min was 0.0042 and the coefficients of the genes were not penalized to 0 (Figure 3A). Next, in the ROC curve, 5 genes (CDC20, CXCL14, FCGR2B, HKDC1, GPBAR1) had an AUC value larger than 0.85 in both GSE38941 and GSE14668, and the values were not all 1. These genes were included as key genes (Figure 3B). Furthermore, in both the GSE38941 and GSE14668 datasets, CDC20, CXCL14, FCGR2B, HKDC1, and GPBAR1 were identified as biomarkers. Their expression levels exhibited significant differences between HBV-ACLF samples and control samples, with consistent expression trends (*p <* 0.05). Notably, CDC20, CXCL14, FCGR2B, HKDC1, and GPBAR1 were all significantly up-regulated in HBV-ACLF samples (Figure 3C).

### 3.5 Nomogram had excellent predictive capability

Based on the five biomarkers, the constructed nomogram was shown in Supplementary Figure 2A. In the calibration curve, the predicted curve was very close to the ideal curve with a p-value of 0.577 in Hosmer-Lemeshow (HL) test (Supplementary Figure 2B). These results showed that the nomogram model had excellent predictive ability for the occurrence of HBV-ACLF.

### 3.6 Enrichment pathways associated with biomarkers were explored

**Figure 4:**
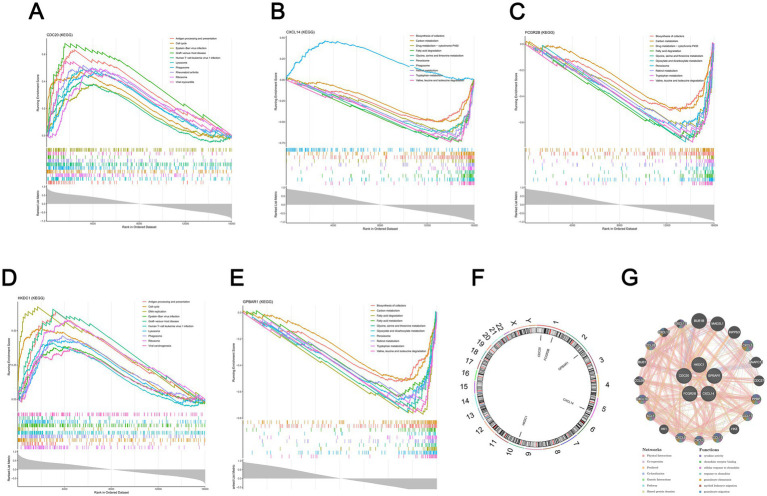
Enrichment pathways associated with biomarkers. **(A)** Top 10 CDC20-associated pathways enriched via GSEA analysis (*p* < 0.05, |NES| > 1, FDR < 0.25). **(B)** Top 10 CXCL14-associated pathways enriched via GSEA analysis (*p* < 0.05, |NES| > 1, FDR < 0.25). **(C)** Top 10 FCGR2B-associated pathways enriched via GSEA analysis (*p* < 0.05, |NES| > 1, FDR < 0.25). **(D)** Top 10 HKDC1-associated pathways enriched via GSEA analysis (*p* < 0.05, |NES| > 1, FDR < 0.25). **(E)** Top 10 GPBAR1-associated pathways enriched via GSEA analysis (*p* < 0.05, |NES| > 1, FDR < 0.25). **(F)** Chromosomal locations of the 5 biomarkers. **(G)** Gene co-expression analysis of the 5 biomarkers using GeneMANIA (https://genemania.org/).

The 150, 197, 203, 142, and 195 pathways were significantly enriched by CDC20, CXCL14, FCGR2B, HKDC1, and GPBAR1, respectively (*p <* 0.05). The top 10 pathways that were significantly enriched by each biomarker were presented, respectively. Specifically, the pathways significantly enriched by CDC20 included cell cycle, phagosome, etc (Figure 4A, Supplementary Table 3). Pathways such as retinol metabolism and carbon metabolism were significantly enriched by CXCL14 (Figure 4B, Supplementary Table 4). As for FCGR2B, the enriched pathways contained tryptophan metabolism, biosynthesis of cofactors, and so on (Figure 4C, Supplementary Table 5). HKDC1 enriched multiple pathways, such as DNA replication and viral carcinogenesis (Figure 4D, Supplementary Table 6). Meanwhile, pathways like biosynthesis of cofactors and carbon metabolism were also significantly enriched by GPBAR1 (Figure 4E, Supplementary Table 7). Subsequently, it was further found that CDC20, CXCL14, FCGR2B, HKDC1, and GPBAR1 were located on chromosomes 1, 5, 1, 10, and 2, respectively (Figure 4F). Additionally, a GGI network was constructed. Genes with functions similar to those of the biomarkers included CXCL5, CXCL12, etc., and the functions involved included cytokine activity, chemokine receptor binding, etc (Figure 4G). The above-mentioned pathways and functions might have played a crucial role in the development of HBV-ACLF.

### 3.7 Biomarkers were regulated by multiple molecules simultaneously

The 55, 44, and 17 TFs targeting CDC20, HKDC1, and GPBAR1, respectively, were predicted. The constructed TF-mRNA network was shown in Supplementary Figure 3A. Among them, KLF13, JUND, and other TFs jointly targeted CDC20 and HKDC1, while ZNF610, TFDP1, and other TFs jointly targeted CDC20 and GPBAR1. Subsequently, miRNAs targeting two biomarkers were predicted, totaling 69 miRNAs. There were 34, 8, 1, 19, 6 and 1 miRNAs targeting CXCL14 and FCGR2B, CXCL14 and HKDC1, CXCL14 and GPBAR1, FCGR2B and HKDC1, FCGR2B and GPBAR1, HKDC1 and GPBAR1, respectively (Supplementary Figure 3B). Then, it was found that a total of 10 miRNAs and 211 lncRNAs were associated. The lncRNA-miRNA-mRNA regulatory network was constructed and the lncRNAs with degree > 1 were demonstrated. Among the interactions included XIST-hsa-miR-296-3p-CXCL14, SNHG14-hsa-miR-510-5p-CXCL14, and so on (Supplementary Figure 3C). To sum up, these discoveries contributed to the methodical disclosure of biomolecule-to-biomolecule interactions and offered crucial hints and a theoretical foundation for comprehending the genesis and progression of HBV-ACLF.

### 3.8 Biomarkers were associated with various diseases and drugs

A disease-mRNA network was constructed. It could be seen that various diseases were associated with the biomarkers. For example, Crohn’s disease and constipation were related to GPBAR1. Notably, carcinoma was associated with CDC20 and CXCL14 (Supplementary Figure 4A). Potential drugs targeting the biomarkers were also predicted, and a drug-mRNA network was constructed. The 10 and 4 drugs targeted FCGR2B and GPBAR1, respectively. Drugs targeting FCGR2B included abciximab, sarilumab, etc., and drugs targeting GPBAR1 included cholic acid, deoxycholic acid, etc (Supplementary Figure 4B). Investigating illnesses and drugs associated with biomarkers contributed to a deeper comprehension of the pathophysiology and development mechanisms of HBV-ACLF, which served as a foundation for early disease diagnosis and prognostic evaluation.

### 3.9 Biomarkers had good binding ability to drugs

**Figure 5:**
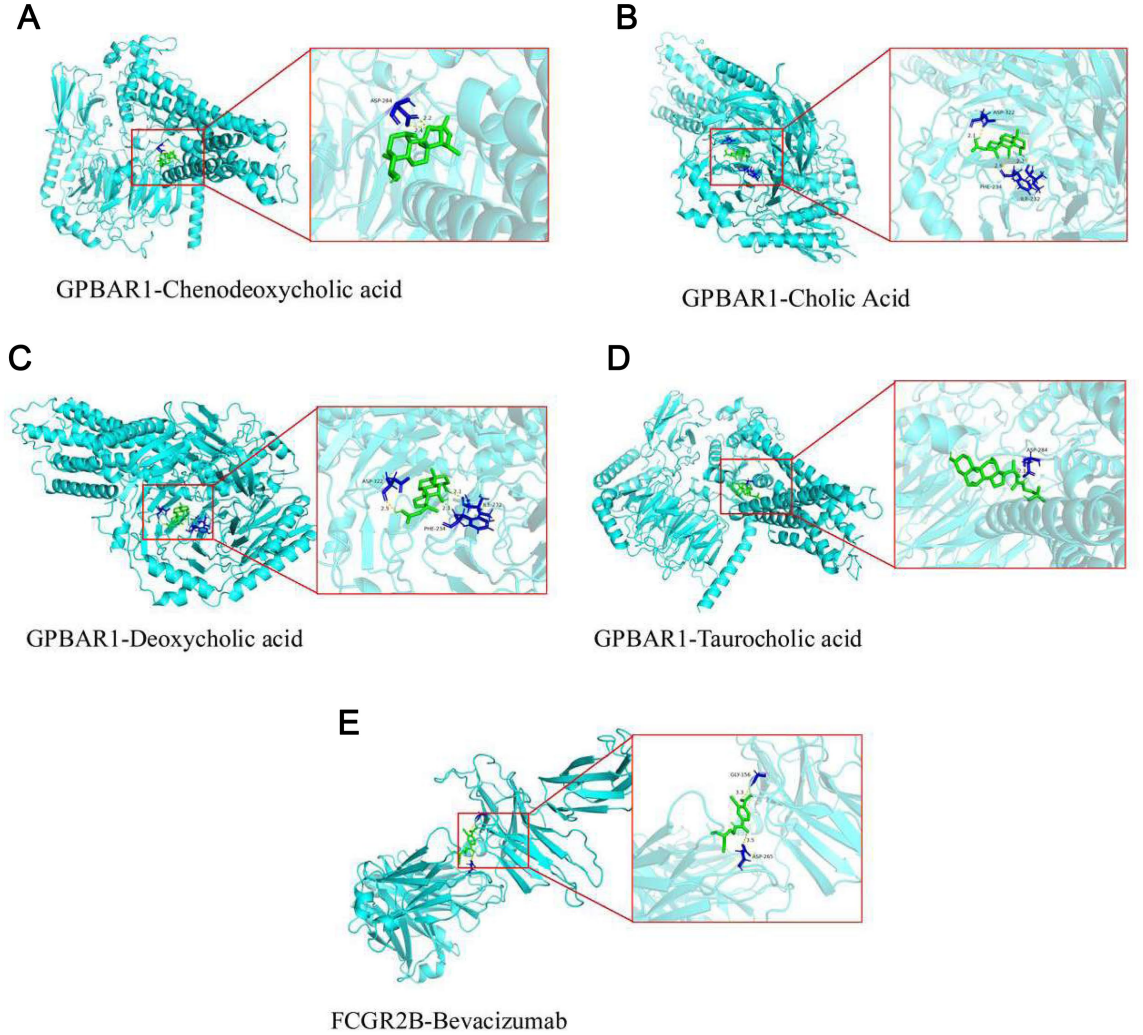
Molecular docking analysis to investigate the binding ability between biomarkers and possible medications. **(A)** The binding between GPBAR1 and chenodeoxycholic acid. **(B)** The binding between GPBAR1 and cholic acid. **(C)** The binding between GPBAR1 and deoxycholic acid. **(D)** The binding between GPBAR1 and taurocholic acid. **(E)** The binding between FCGR2B and bevacizumab.

The predicted drugs were, respectively, subjected to molecular docking with the corresponding biomarkers. The results showed that the binding free energy between GPBAR1 and chenodeoxycholic acid was −9.5 kJ/mol, binding through the residue ASP-284 (Figure 5A). The binding free energy between GPBAR1 and cholic acid was −7.5 kJ/mol, binding through the residues ASP-322, PHE-234, and ILE-232 (Figure 5B). The binding free energy between GPBAR1 and deoxycholic acid was −8.3 kJ/mol, binding through the residues ASP-322, PHE-234, and ILE-232 (Figure 5C). The binding free energy between GPBAR1 and taurocholic acid was −8.0 kJ/mol, binding through the residue ASP-284 (Figure 5D). The binding free energy between FCGR2B and bevacizumab was −5.0 kJ/mol, binding through the residues GLY-156 and ASP-265 (Figure 5E). All these results indicated that the biomarkers had good binding ability with the potential drugs.

### 3.10 Validation of biomarkers

**Figure 6:**
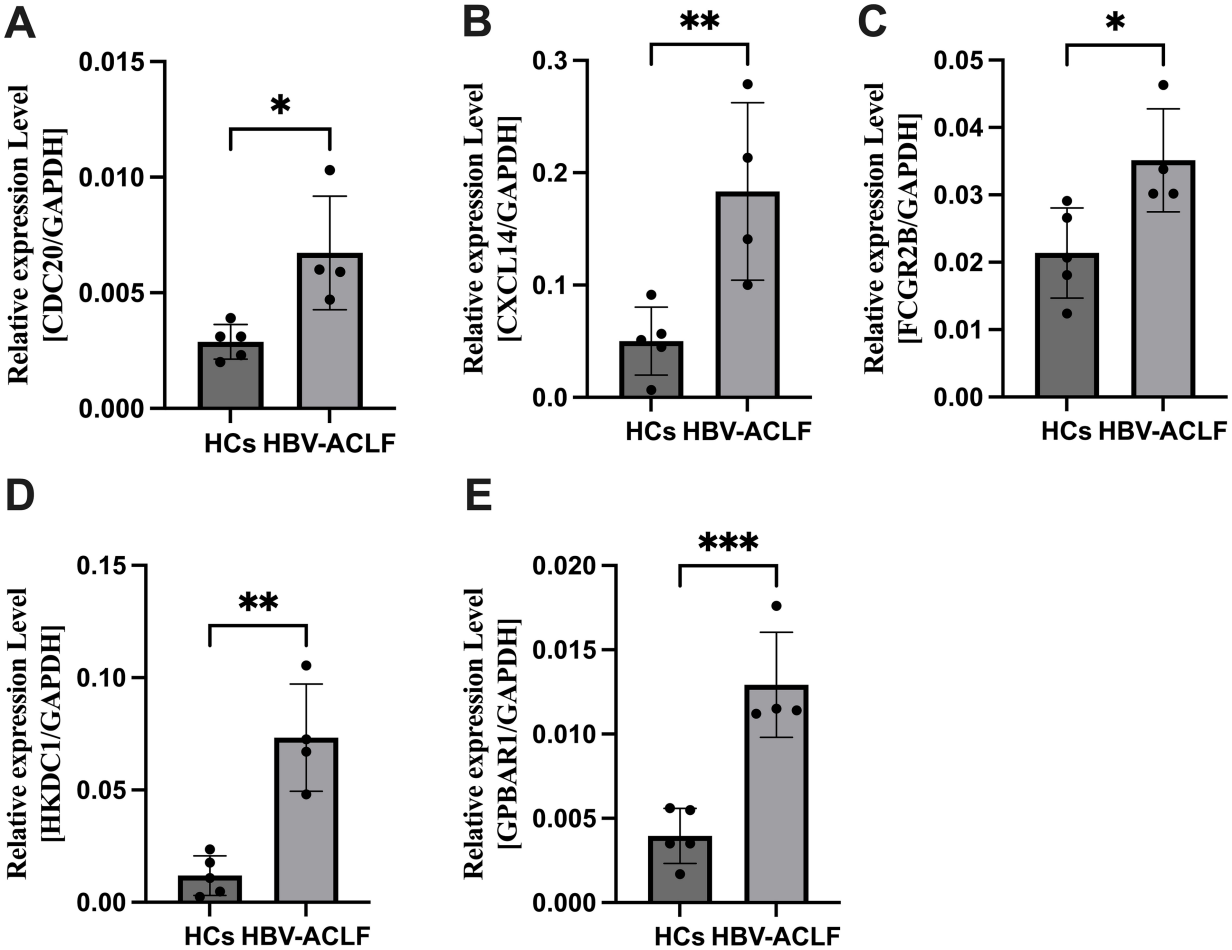
The expression level of CDC20, CXCL14, FCGR2B, HKDC1, GPBAR1 in liver tissue samples between HBV-ACLF patients and healthy controls (HCs). **(A)** The expression level of CDC20 between HBV-ACLF patients and HCs. **(B)** The expression level of CXCL14 between HBV-ACLF patients and HCs. **(C)** The expression level of FCGR2B between HBV-ACLF patients and HCs. **(D)** The expression level of HKDC1 between HBV-ACLF patients and HCs. **(E)** The expression level of GPBAR1 between HBV-ACLF patients and HCs. *p < 0.05, **p < 0.01, ***p < 0.001.

Total RNA was extracted from liver tissue samples of HCs and HBV-ACLF patients. Five genes identified as potential candidate biomarkers for HBV-ACLF were validated using qRT-PCR. The expression levels of CDC20, CXCL14, FCGR2B, HKDC1, and GPBAR1 were significantly upregulated in HBV-ACLF patients compared with HCs (*p* < 0.05; Figure 6).

## 4 Discussion

In this study, we identified five genes (CDC20, CXCL14, FCGR2B, HKDC1, and GPBAR1) as diagnostic biomarkers for HBV-ACLF, highlighting their roles in M1 macrophage polarization and bile acid signaling. The identification of these novel biomarkers in HBV-ACLF reveals previously underrecognized pathways involving cell cycle regulation, metabolic dysfunction, and bile acid–mediated immune modulation. These findings expand the current immune-centered model of HBV-ACLF pathogenesis and suggest novel targets for biomarker development and therapeutic intervention.

These novel biomarkers reflect distinct yet interconnected pathways that corroborate the complex interplay of immune and metabolic processes during the development and progression of HBV-ACLF. CDC20, a regulator of the cell cycle, is upregulated in several liver diseases and may drive hepatocyte apoptosis by destabilizing the anaphase-promoting complex (Zhao et al., 2021). CXCL14, a chemokine linked to macrophage recruitment, aligns with the observed M1 polarization in liver failure. Circulating CXCL14 levels were established as a novel early prognostic biomarker for poor outcomes in patients with acetaminophen-induced acute liver failure (Umbaugh et al., 2024). FCGR2B, an inhibitory Fcγ receptor, paradoxically demonstrated elevated expression in this study, implying compensatory anti-inflammatory feedback in HBV-ACLF (Zhou et al., 2025). HKDC1, a target of TFEB, plays roles in glucose metabolism and is essential for maintaining both mitochondrial and lysosomal homeostasis (Cui et al., 2024). Meanwhile, GPBAR1 functions as a dual modulator of bile acid signaling and macrophage polarization (Shi et al., 2020; Liu et al., 2025). Previous studies revealed that circulating bile acids can induce immunosuppression in septic shock patients with severe liver failure (Leonhardt et al., 2023).

Our findings bridge two understudied aspects of HBV-ACLF: macrophage dominance and bile acid receptor signaling. The enrichment of tryptophan metabolism pathways, a known modulator of macrophage polarization, supports the hypothesis that macrophage polarization in HBV-ACLF is metabolically driven (Liu et al., 2024). GPBAR1’s involvement in this network suggests that bile acids may directly or indirectly prime macrophages toward a pro-inflammatory phenotype. Furthermore, the lncRNA-miRNA-mRNA network (e.g., XIST/miR-296-3p/CXCL14) reveals post-transcriptional regulation of M1 polarization, offering targets for RNA-based therapies.

The diagnostic nomogram incorporating these biomarkers showed good diagnostic efficacy. Clinically, this could enable earlier intervention in high-risk patients. Drug prediction and docking analyses further highlight potential therapeutic targets for HBV-ACLF. Additionally, the SNHG14/miR-510-5p/CXCL14 axis provides a rationale for targeting lncRNAs with antisense oligonucleotides, a strategy being explored in cancer but not yet investigated in HBV-ACLF.

This study presents both notable strengths and limitations. Through comprehensive multi-omics profiling, we identified five key genes (CDC20, CXCL14, FCGR2B, HKDC1, and GPBAR1) that play critical roles in cell cycle regulation, metabolic dysfunction, and bile acid-mediated immune modulation, highlighting their potential as diagnostic biomarkers or therapeutic targets for HBV-ACLF. However, several limitations should be acknowledged. First, our reliance on bulk RNA sequencing may obscure cell-type-specific transcriptional dynamics, which could be better resolved through single-cell RNA-seq analysis of HBV-ACLF liver tissues. Second, the precise mechanistic roles of these genes in macrophage polarization remain to be fully elucidated, necessitating further validation in cellular and animal models. Additionally, protein-level confirmation (e.g., Western blot or immunohistochemistry) would strengthen the biological relevance of our findings. Finally, while our study provides valuable insights, the clinical cohort size was limited due to the challenges in prospectively collecting liver tissue samples from HBV-ACLF patients. Future multi-center, large-cohort studies are warranted to validate and extend our findings.

In summary, this study establishes CDC20, CXCL14, FCGR2B, HKDC1, and GPBAR1 as key biomarkers in HBV-ACLF. By integrating bioinformatics with translational validation, we propose a biomarker-driven framework for diagnosis and therapy of HBV-ACLF, bridging the gap between molecular insights and clinical management of this lethal syndrome.

## Data Availability

The original contributions presented in the study are included in the article/Supplementary material, further inquiries can be directed to the corresponding author/s.

## Footnotes

^1^ https://www.ncbi.nlm.nih.gov/gds

^2^ https://string-db.org/

^3^ http://www.genemania.org

^4^ https://www.encodeproject.org/

^5^ https://www.networkanalyst.ca/

^6^ http://mirwalk.umm.uni-heidelberg.de/

^7^ http://starbase.sysu.edu.cn

^8^ http://ctdbase.org/

^9^ https://go.drugbank.com/

^10^ http://www.rcsb.org/pdb/

^11^ https://pubchem.ncbi.nlm.nih.gov/

^12^ https://autodock-vina.readthedocs.io/en/latest/index.html
