# Supplementary figures and images for: Multi-omics profiling identifies M1 macrophage polarization-associated biomarkers in hepatitis B virus-related acute-on-chronic liver failure
Source: Front Microbiol. 2025 Sep 24;16:1630042. doi: 10.3389/fmicb.2025.1630042 (PMC12504312; doi:10.3389/fmicb.2025.1630042)

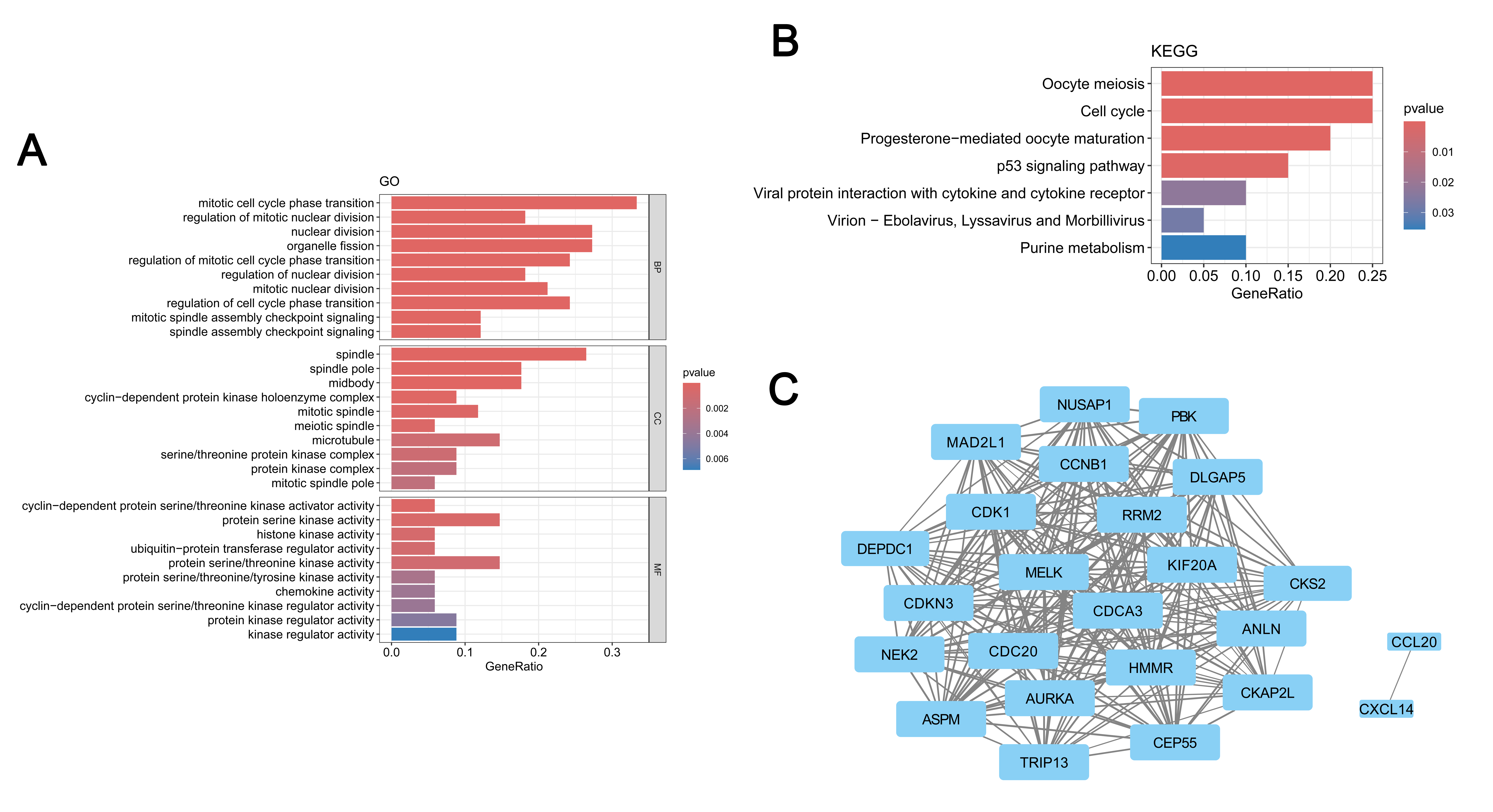

Supplement: Supplementary file 1 [file Image_1.tif]

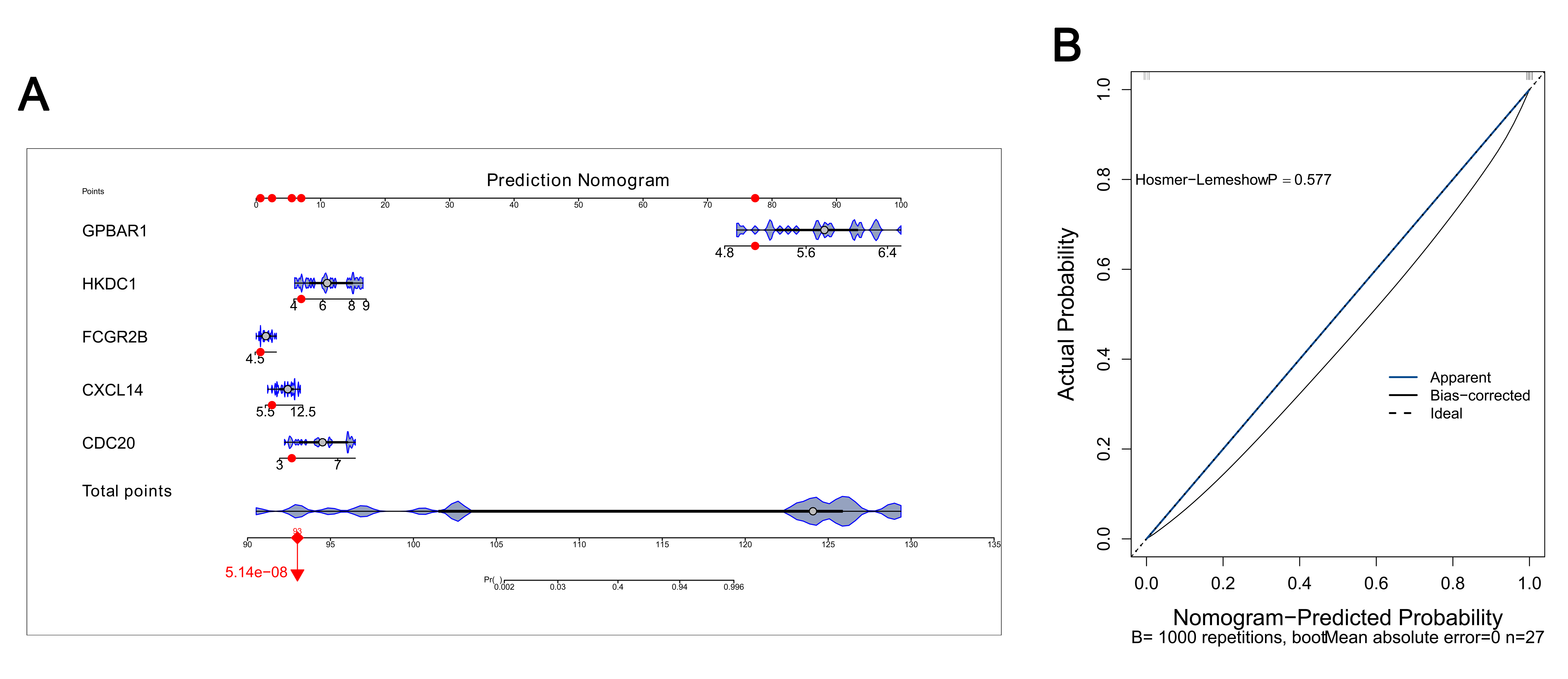

Supplement: Supplementary file 2 [file Image_2.tif]

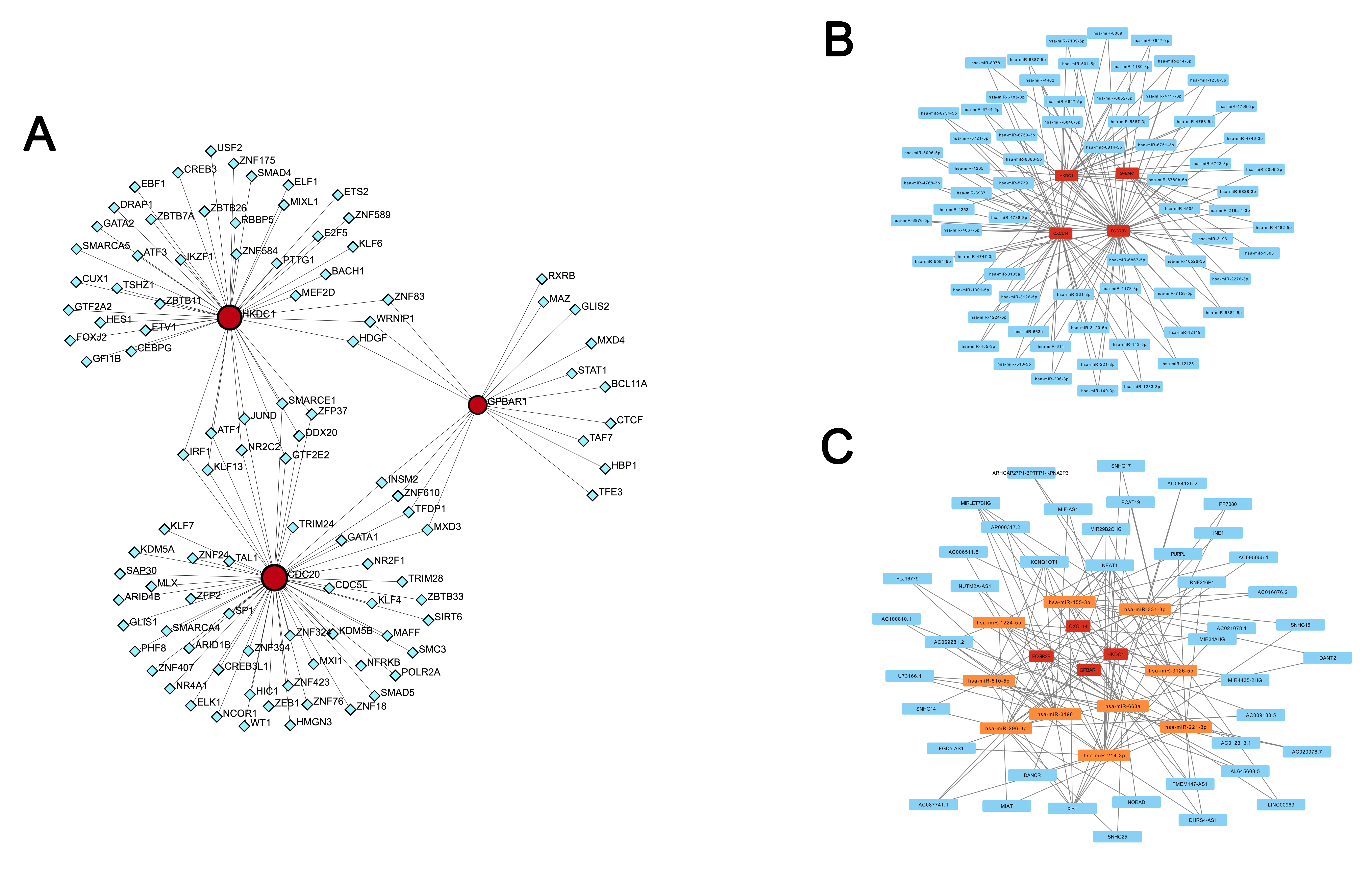

Supplement: Supplementary file 3 [file Image_3.tif]

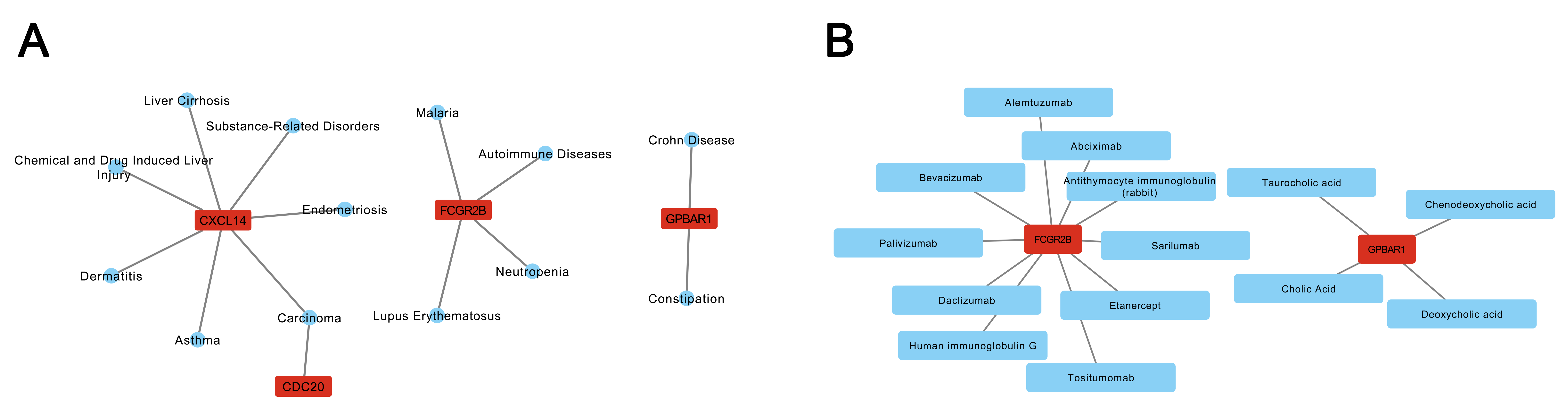

Supplement: Supplementary file 4 [file Image_4.tif]
